# Identification of a growth factor required for culturing specific fastidious oral bacteria

**DOI:** 10.1080/20002297.2022.2143651

**Published:** 2022-11-25

**Authors:** Pallavi Murugkar, Eric Dimise, Eric Stewart, Stéphane N. Viala, Jon Clardy, Floyd E. Dewhirst, Kim Lewis

**Affiliations:** a Antimicrobial Discovery Center, Department of Biology, Northeastern University, 134 Mugar Hall, 360 Huntington Ave 02115, Boston, MA, USA; b Department of Biological Chemistry and Molecular Pharmacology, Harvard Medical School 02115, Boston, MA, USA; c Department of Microbiology, the Forsyth Institute, Cambridge, MA, USA; d Department of Oral Medicine, Infection and Immunity, Harvard School of Dental Medicine, Boston, MA, USA

**Keywords:** *Porphyromonas pasteri*, menaquinone, 1,4-dihydroxy-2-naphthoic acid (DHNA), bacterial cultivation, growth factors, genomic analysis

## Abstract

**Aims:**

The aim of this research was to isolate oral bacteria that are dependent for growth on adjacent bacteria producing a required growth factor and to identify the chemical structure of the growth factor.

**Methods:**

*Porphyromonas pasteri* strain KLE1280, could be cultivated with *Staphylococcus hominis* and *Escherichia coli* as helper strains. A deletion mutant library of E. coli was screened to determine genes involved in production of the growth factor. Compounds produced by the growth factor’s pathway were screened to see if they would stimulate growth of strain *P. pasteri* KLE1280. The genomes of species related to *P. pasteri* KLE1280 were screened for presence of the factor’s synthetic pathway.

**Results:**

Analysis of the *E. coli* deletion mutant library and growth studies identified 1,2-dihydroxy-2-naphthoic acid (DHNA) and menaquinone-4 (MK4) as the growth factors. Strain *P. pasteri* KLE1280 was shown to lack five genes in the menaquinone synthesis pathway but to possess the two genes necessary to convert DHNA to menaquinone. Genome analysis found that 8 species in genera *Porphyromonas* and *Tannerella* lack five genes in the menaquinone synthesis pathway.

**Conclusions:**

Addition of DHNA to culture media allows isolation of strains of several oral species that are not recovered using standard media

## Introduction

Bacteria that have never been cultured in the laboratory are found in almost every environment, including the human body [1–4]. This uncultivability presents a significant barrier to investigating the properties of these bacteria and understanding their role in health and disease. A common cause of ‘uncultivability’ in environments such as soil or marine sediment is the dependence of uncultured bacteria on growth factors produced by cultivable neighboring species [5]. We hypothesized that a similar relationship may exist between uncultured bacteria of the oral microbiome and their neighbors. For example, siderophores, iron-chelating compounds produced by bacteria, have been shown to facilitate growth of difficult-to-culture oral species *Fretibacterium fastidiosum* and *Prevotella* sp. HMT-376 [6–8]. The identification of growth factors such as siderophores has proven useful for cultivation of several fastidious species of oral bacteria in addition to the species they were initially identified as helping.

Dental plaque, a biofilm with a complex structure [9–11], is an excellent source of bacteria that require growth factors from their neighbors. The majority of oral taxa in the Human Oral Microbiome Database have reduced genomes of under 3 Mb, and many have known auxotrophies for certain vitamins, amino acids, lipids, and cell wall components [12,13]. Rich media often contain peptone, yeast extract, and blood to provide commonly needed growth factors. However, some fastidious bacteria are auxotrophic for as yet unknown factors not contained in these rich media and therefore can only be grown in coculture with other bacteria. To complement such auxotrophies, cross-streaking a culture plate with a helper bacterium has been used successfully to isolate and grow several dependent oral bacteria [6–8,14,15]. Identification of the factors commonly produced by such helper bacteria would improve understanding of taxon–taxon relationships in complex biofilms and would enable supplementation of cultivation media so that fastidious taxa could be isolated in pure culture.

Currently, 28% of the oral taxa in the Human Oral Microbiome Database remains uncultured [13]. While some of these taxa may not have been cultured because they have low abundance in the microbiome or low prevalence in the population, others have not yet been cultured because of their unusual auxotrophies. The aim of this study was to identify fastidious bacteria dependent on other bacteria for growth and then identify the specific growth factor involved in the dependence. Using both manual plating and cell sorting, we identified dental plaque bacteria dependent on adjacent helper bacteria for growth. We then identified the auxotrophy for one such dependent strain, *Porphyromonas pasteri* strain KLE1280, using an *Escherichia coli* mutant library screen as well as genomic analysis and *in vitro* validation of results. Our findings suggest that addition of 1,4-dihydroxy-2-naphthoic acid (DHNA) to culture media would improve the growth and recovery of fastidious taxa including multiple strains from the genus *Porphyromonas.*

## Materials and methods

### Culture media

Trypticase Soy Broth (TSB), Trypticase Soy Agar (TSA), Luria-Bertani broth (LB), and Luria-Bertani Agar (LBA) Bacto^TM^ were obtained from ThermoFisher. Brain Heart Infusion Broth (BHI), Brain Heart Infusion Agar (BHIA), R2A broth (R2A), and R2NP broth (R2NP) were obtained from Difco. Columbia Blood Base Agar (CBBA) was obtained from Becton Dickinson. Fastidious Anaerobe Broth (FAB) and Fastidious Anaerobe Agar (FAA) were obtained from Neogen. FAABS was comprised of FAA with 5% sheep blood and 5% pooled human saliva. Chocolate Blood Agar (CBA) was comprised of TSA with 5% sheep blood. Modified Fluid Medium (mFUM) was made from reagents according to Guggenheim [16].

### Subjects and sample collection

The goal of this study was to identify commensal helper-dependent pairs. To this end, dental plaque was collected from six healthy individuals, self-reported to be healthy, who had not had antibiotic treatment in the 6 months prior to sample collection. The study was conducted under Northeastern University Institutional Review Board (IRB) approval #08-11-15. Informed consent was obtained from all subjects.

Subjects collected supragingival plaque on sterile toothpicks, which were dropped into tubes with 1 mL R2NP medium. Plaque was dislodged from the toothpick by rotation against the tube wall and the tube vortexed vigorously to disperse cells. The plaque suspension in broth was processed for culture immediately and separately for each of the six subjects.

### Anaerobic culture

Manipulations (serial dilutions and plating) were done in room air on the bench as quickly as possible (less than 30 min) and the plates were incubated anaerobically as soon as manipulations were completed. All bacterial cultures were incubated in an anaerobic chamber (Coy Lab Products) at 37°C with an atmosphere comprised 5% H_2_, 10% CO_2_, and 85% N_2_. A palladium catalyst was used to scour oxygen.

#### Identifying colonies whose growth is dependent on adjacent colonies

##### Manual approach

Suspended plaque samples were serially diluted 10-fold in broth media, and 100 µL of each dilution spread onto R2A, TSA, LBA, BHIA, BA, CBA, FAA and FAABS media. Plates were incubated anaerobically at 37°C for 3–7 days. The plates were checked daily for the presence of small or late appearing colonies. Small colonies (diameter <1 mm) growing close to large colonies (diameter >1 mm) on plates that had 100–300 colonies were identified as potential dependent and helper colony pairs.

##### Cell sorting approach

One hundred µL of dental plaque was diluted 1:1,000 in phosphate buffered saline (PBS). The plaque was vortexed vigorously to break visible clumps and pipetted up and down multiple times to homogenize the sample. Cells were then sorted with a BD FACS Aria II cell sorter and directly deposited by the cell sorter onto arrays of either 24, 96, or 384 cells on FAABS, mFum, and CBA media in Omni trays (rectangular one-well plates, 127 mm × 85 mm). This allowed for a defined distance between cells on a plate. As the number of spots increased on one plate, the distance between the colonies decreased. All plates were incubated anaerobically at 37°C for 3–7 days. These plates were also checked daily for the presence of small or late appearing colonies, which were identified as potential growth-dependent colonies surrounded by potential helper colonies in the square array. A helper mix was created by pooling the eight colonies surrounding a potential-dependent colony. In a study of the effect of distance to neighboring colonies on recovery of isolates, cells were sorted onto plates of mFum agar in arrays of 24, 96, 384 cells and also a 96 well plate with mFum broth.

### Validating growth dependence

Potential dependent colonies and their helpers were subcultured on the same medium from which they were picked. The small colony and its adjacent large colony (manual approach) or the small colony and a mix of the adjacent arrayed colonies (cell-sorting approach) were transferred. The small colony was spread evenly on the entire plate, and the helper or helper mix was spotted off center. Dependence was initially verified by growth of cells satelliting the helper colony, but not growing independently elsewhere on the plate. The portion of the plate far from the helper spot served as a negative control for growth. Dependence was further validated by lack of independent growth on any of the eight media used in the manual approach.

### Frozen stocks

Dependent isolates and helper strains or helper mixes were stored at −80°C as 15% glycerol stocks in the medium from which they were picked.

### Identification of isolates by 16S rRNA gene sequencing

Those isolates that were selected for further analysis and their helpers were characterized by 16S rRNA gene sequencing using the universal primers 27 F and 1492 R, which amplify a 1,466 bp region of the 16S rRNA gene [17]. The isolates were identified using the EZ-Taxon server (http:// www.eztaxon.org/) [18], by BLAST analysis using the Human Oral Microbiome Database (HOMD) (http://www.homd.org) [1] and by BLAST analysis of the NCBI rRNA/ITS database.

### *Identification of* E. coli *genes required for stimulating* P. pasteri *KLE1280 growth*

Libraries of deletion mutants of *E. coli* K-12 MG1655 with non-essential genes deleted (single and multiple) were ordered from the Keio collection [19,20]. A set of 310 deletion mutants, collectively representing deletions of most of the non-essential genes of *E. coli*, were picked and reorganized into a smaller library. Thirty-five microliters of 10^7^ cells/ml of *P. pasteri* KLE1280 from a glycerol frozen stock was spread onto FAABS plates, and each plate was spotted with 5 µL of a deletion mutant. Plates were incubated anaerobically at 37°C for 3 days. Results were noted as presence and absence of growth induction around the helper.

### *Whole-genome sequencing of* P. pasteri *KLE1280*

Whole-genome sequencing was performed at the Genome Institute at Washington University, St. Louis, MO, using Illumina sequencing. The draft genome was annotated using the RAST (Rapid Annotations using Subsystems Technology) server [21] and Tigra assembler [22]. Presence of genes was determined by the detection of open reading frames (ORF).

### Quinone isolation and purification or source

Quinones MK4, Q1, Q2, Q4, Q9 and Q10 were obtained from Sigma, Q8 and MK8 were extracted and purified from *E. coli* and MK4, MK5, MK6, MK7 and MK8 were purified from *Micrococcus luteus*. Cells of *E. coli or M. luteus* were resuspended in 50 mL 3:2:1 ethanol, H_2_O, 25% sodium hydroxide. The cell suspension was then refluxed under inert atmosphere (argon) for 20 min at 100°C. The vessel was immediately cooled in an ice bath. The contents were then poured into a separating funnel and extracted three times with heptanes (~200 mL portions). The organic layers were collected, rinsed with brine and dried with anhydrous sodium sulfate. They were then completely dried in a rotary evaporator. The dried material was stored at −20°C under Argon gas until use. An Agilent Technologies 1200 Series High-Performance Liquid Chromatography system equipped with G1361A Prep Pumps and a G1315D diode array detector was used for purification of quinones, and Nuclear Magnetic Resonance (NMR) spectroscopy was used to elucidate the structure of quinones as described previously [5,23].

### *Testing addition of quinones for stimulating growth of* P. pasteri *KLE1280*

Ubiquinones Q1, Q2, Q4, Q8, Q9, Q10 and menaquinones MK4, MK5, MK6, MK7, and MK8 were mixed with warm FAABS at final concentrations of 5 µg/mL, and 200 µL of the warm agar was added to wells of a 96-well plate. Ten microliters of *P. pasteri* KLE1280 at various cell densities (diluted in FAB from glycerol stocks) was added on top of the solidified agar, and the plates were incubated anaerobically at 37°C for 3 days. The menaquinone biosynthesis pathway intermediate DHNA (Sigma) was also tested.

### *Heme requirement of* P. pasteri *KLE1280*

FAA agar, as prepared by the manufacturer, contains both hemin and menadione. For these experiments, we prepared FAA from individual components without adding hemin and menadione. The heme requirement of *P. pasteri* KLE1280 was determined by preparing FAA agar both unsupplemented and supplemented with blood (5%), hemin (10 µg/mL) or hemoglobin (100 µg/mL). These plates were then streaked with 100 µL of glycerol-frozen stock and spotted with 10 µL of various concentrations of MK4 (0.01 mg/mL, 0.1 mg/mL, 1 mg/mL, 10 mg/mL). The plates were incubated anaerobically at 37°C for 3 days.

### *Testing grow dependence of other* Porphyromonas *sp. on menaquinones*

*P. pasteri* strains were maintained in the anaerobic chamber on FAA supplemented with 10 µg/mL DHNA (Sigma Aldrich). Colonies were harvested directly from FAA and spread on CBBA plates that had been pre-reduced in the anaerobic chamber. Plates were allowed to dry; a 1 µL spot of either 95% ethanol, 10 µg/µL DHNA in 95% ethanol or 10 µg/µL menadione in 95% ethanol was applied to the plate in an off-center position. The plates were incubated anaerobically, and growth was recorded photographically on days 1 and 3.

*Porphyromonas gingivalis* strains were maintained in the anaerobic chamber on FAA supplemented with 10 mg/L DHNA. Colonies were harvested directly from plates and spread on BHIA medium supplemented with 10 mg/L hemin (Sigma Aldrich). Plates were allowed to dry and then spotted with 1 µL of either 95% ethanol, 10 µg/µL DHNA in 95% ethanol, or 10 µg/µL menadione in 95% ethanol. The plates were incubated anaerobically, and growth was recorded photographically on days 1 and 3.

## Results

### The effect of proximity to neighboring bacteria on recovery of isolates

To determine if proximity of cells on a plate influenced the recovery of bacteria, dental plaque samples were dispersed and sorted for single cells, which were plated into a 96 well plate with mFum broth or plated onto one-well mFum agar plates in arrays of 24, 96 or 384 spots. As the number of spots increases on the one-well Omni tray, the distance between the spots, and resultant colonies, decreases. This allows for diffused growth factors produced by potential helpers to reach a greater number of dependents due to lesser distances between colonies on the plate. This is reflected in the results as shown in Table 1; cells with no neighbors in 96-well broth plates had the lowest recovery. When sorted onto plates, the recovery increased with cell density.

**Table 1.** The effect of proximity to neighboring bacteria on colony recovery.

Density	Recovery	% Recovery
384 cells	137/384	36
96 cells	129/384	34
24 cells	109/384	28
96 cells (1 cell per well)	105/384	27

### Identifying bacteria whose growth is dependent on adjacent bacteria

**Figure 1.**
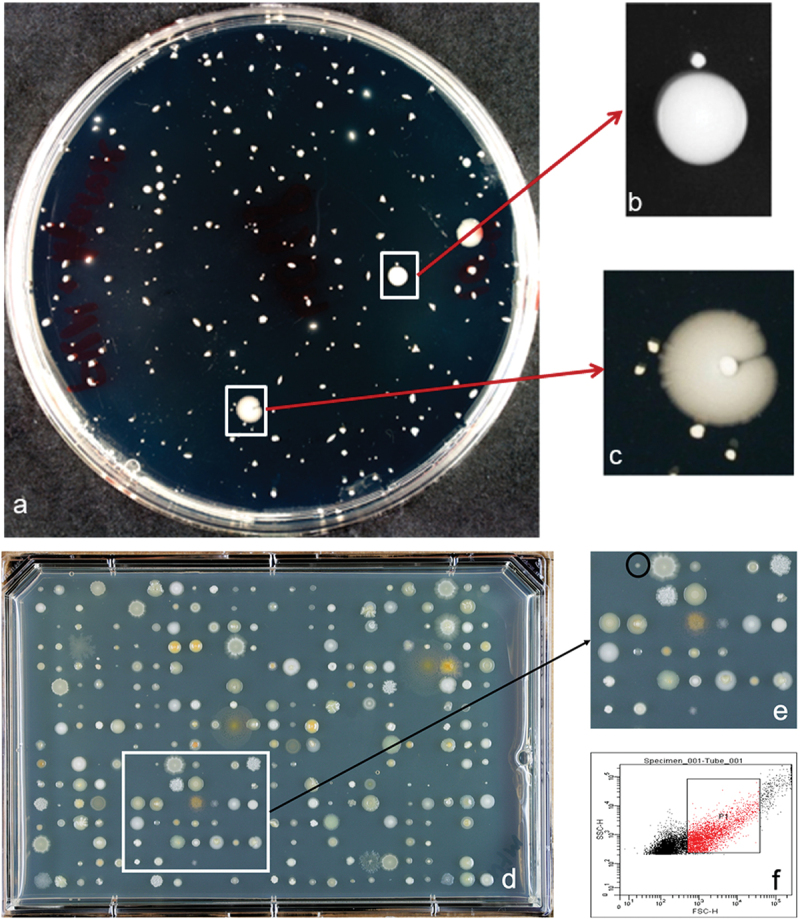
Screening for uncultured isolates. (**a**) Serial dilution spread plate of bacteria from dental plaque. (**b, c, e**) Small colonies growing in proximity of larger colonies. These small colonies were picked and either spread or streaked onto fresh media plates for identifying dependent bacteria. (**d**) Cell sorting plate with 384 events from dental plaque sorted onto the plate. (**f**) The selected population (P1) from dental plaque for sorting.

In the initial screen for growth factor-dependent strains, manual dilution plates were examined for the presence of small or late-appearing colonies growing adjacent to larger colonies. The image of a typical plate is shown in Figure 1a with enlargements of small colonies satelliting larger colonies shown in Figure 1b,1c. A plate of single bacterial cells sorted and spotted in arrays is shown in Figure 1d with an enlargement showing two small colonies in Figure 1e. A typical selected plaque cell population for single-cell arraying is shown in Figure 1f.

**Figure 2.**
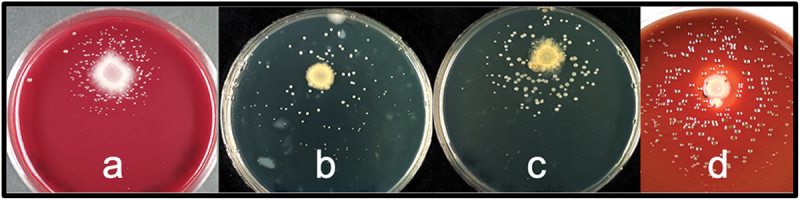
Dependent isolates growing in small colonies around the spotted helper mix. Dependent strains were spread on the entire plate and helper/helper mix was spotted. The dependent strains were identified as (**a**) *Eubacterium yurii*, (**b**) *Prevotella oulorum*, (**c**) *Prevotella tannerae* and (**d**) *Porphyromonas pasteri.*

Subculture validation of potential growth factor-dependent isolates spread on plates with spotted helper cells is shown in Figure 2. The strategy of picking adjacent colonies of a potentially dependent bacterium and adjacent helpers led to the successful isolation and validation of seven dependent oral bacterial strains. These dependent strains passed all validation steps for dependent growth and the strains were identified as *Eubacterium yurii* subsp. *yurii* ATCC 43713 ^T^ (99.6% identical in the sequenced region of the 16S rRNA gene), *Lautropia mirabilis* AB2188^T^ (99.0%), *Peptostreptococcus massiliae* 2002–69,396 (99.2%), *P. pasteri* F0450 (99.6%), *Prevotella oulorum* ATCC 43324 ^T^ (99.7%), *Prevotella tannerae* ATCC 51259 ^T^ (99.0%), *Solobacterium moorei* JCM 10645 ^T^ (99.2%), and *Candidatus Peptostreptococcus massiliae* 2002–69,396 (99.2%).

### *Characterizing the growth dependence for* P. pasteri *KLE1280*

**Figure 3.**
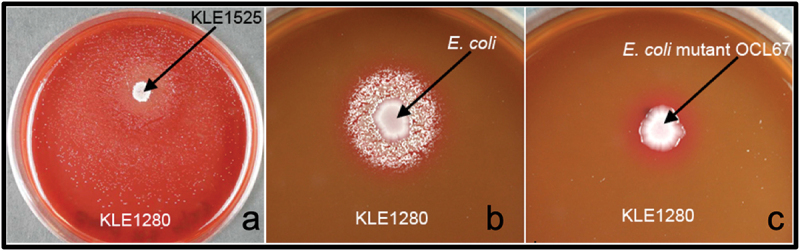
Growth induction of *Porphyromonas pasteri* KLE1280 grown on FAASB plates. (**a**) Growth induction by initial helper *Staphylococcus hominis* KLE1525. (**b**) Growth induction by *E. coli* K-12. (**c**) Lack of growth induction by deletion mutant OCL67.

**Figure 4.**
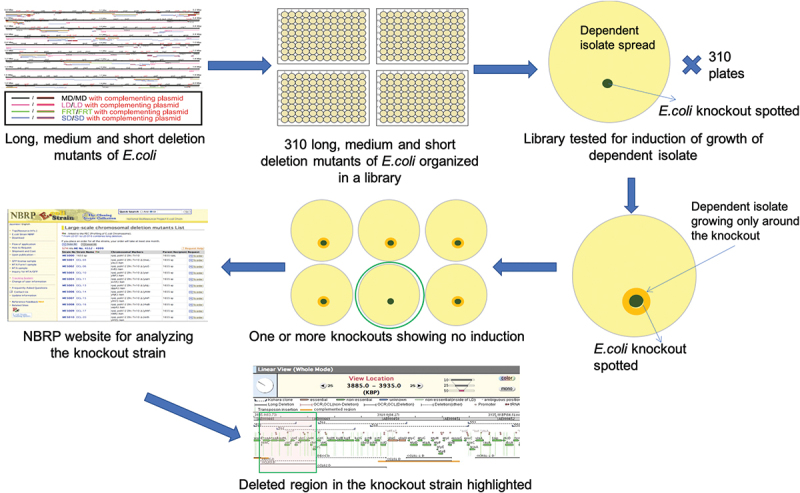
Genetic screen using *Escherichia coli* deletion mutants. A library of some of the long, medium and short deletion mutants of *E. coli* covering many of the non-essential genes was assembled from available libraries. These strains were tested for growth induction of the dependent isolate. The mutant that did not show growth induction of the dependent isolate was subsequently analyzed further.

**Figure 5.**
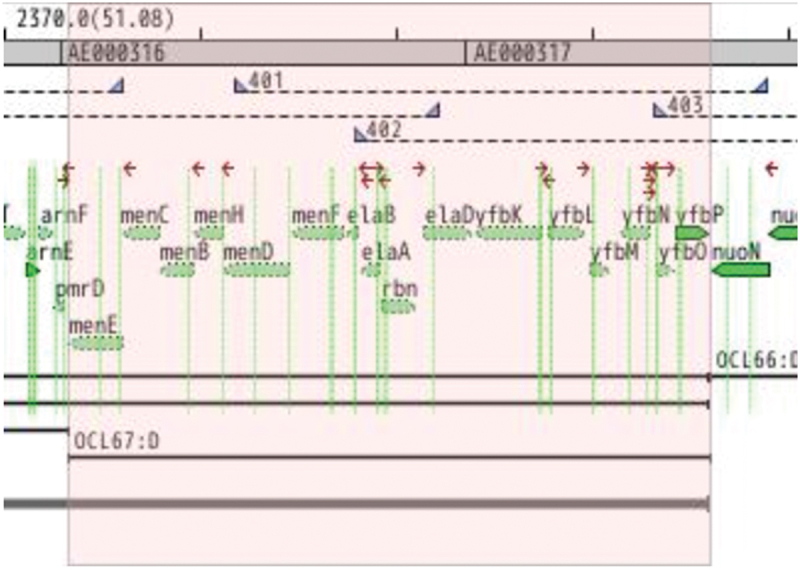
Genes deleted in *E. coli* deletion mutant OCL67 are those shown in the shaded region. Five genes in the menaquinone synthesis pathway are in this region: menE, menC, menB, menH, and menD.

**Figure 6.**
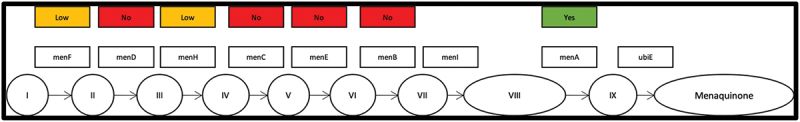
Menaquinone biosynthesis pathway in *E. coli*. (I) Chorismate (II) Isochorismate (III) 2-succinyl-5-enolpyruvyl-6-hydroxy-3-cyclohexene-1-carboxylate (IV) (1 *R*,6 *R*)-6-hydroxy-2-succinylcyclohexa-2,4-diene-1-carboxylate (V) *o*-succinylbenzoate (VI) *o*-succinylbenzoyl-CoA (VII) 1,4-dihydroxy-2-naphthoyl-CoA (VIII) 1,4-dihydroxy-2-naphthoate (IX) demethylmenaquinol-8. Deletion mutants in all known steps were tested for induction of growth of *P. pasteri* KLE1280 (top row boxes). *E. coli* mutants *ΔmenD, ΔmenC, ΔmenE* and *ΔmenB* did not induce growth of *P. pasteri* KLE1280.

Identification of the chemical structure of a growth factor for a microbe allows for the chemical’s addition to culture media to support the axenic growth of that bacterium and other bacteria with the same auxotrophy. For this reason, we chose to characterize the growth factor required for one of our isolates, *P. pasteri* KLE1280. In the initial screen, it was found to depend on *Staphylococcus hominis* KLE1525 (Figure 3a) for growth, but further tests showed that *E. coli* strain K-12 could also support its growth (Figure 3b). This opened the possibility of using a genetic screen to rapidly identify a possible growth factor produced by *E. coli*. An overview of the genetic screen is shown in Figure 4. From the available deletion libraries for *E. coli* K-12 [19,20,24], we assembled a set of 310 deletion mutants, which covered most non-essential *E. coli* genes. Among these, the *E. coli* deletion mutant OCL67 was unable to support growth of *P. pasteri* KLE1280 (Figure 3c). *E. coli* OCL67 is missing 16 genes, including six that make up the menaquinone biosynthesis operon (*menBCDEFH*) (Figure 5). Most of the other *E. coli* mutants that were impaired in growth induction contained deletions related to the biosynthesis of menaquinone or chorismate. Chorismate is a precursor for menaquinone biosynthesis, and a gene deletion mutant in chorismate biosynthesis would consequently result in the absence of menaquinone production. We then tested 16 strains from the KEIO collection, each carrying a deletion in one of the 16 genes deleted in OCL67. We also tested additional mutants deleted in the menaquinone or ubiquinone biosynthetic pathways. Most Δ*men* strains were impaired in inducing growth of *P. pasteri* KLE1280, while the ten strains with deletions in the other genes were able to fully induce growth. *E. coli* mutants with deletions in the last two genes in the menaquinone pathway, *menA* or *ubiE* were able to support growth. The menaquinone synthesis pathway and the gene deletion results are shown in Figure 6.

Editor’s note: Parts of Figure 4 could be difficult to read

### *Ability of quinones to stimulate growth of* P. pasteri *KLE1280*

Based on the finding that disruption of the menaquinone synthesis pathway in *E. coli* led to loss of growth stimulation, we screened products and intermediates of the menaquinone pathway and other selected quinones for their ability to support the growth of *P. pasteri* KLE1280. The results of this screen are shown in Table 2. DHNA stimulated the growth of *P. pasteri* KLE1280. MK4 promoted growth, but not MK5 – MK8, menaquinones with longer isoprenyl side chains. The failure is likely due to lack of these hydrophobic compounds escaping from the cell membrane and diffusing in the assay medium [25]. *P. gingivalis* and *Tannerella forsythia* are known to make very hydrophobic menaquinones MK9, MK10 and MK11 [26,27]. The failure of menadione at 5 µg/mL to stimulate growth of *P. pasteri* KLE1280 in the initial screen is not unexpected even though menadione is often added to culture media for fastidious bacteria such as FAA medium which contains 1 µg/mL. Menadione totally lacks the isoprenyl side chain and therefore is not expected to be partitioned into the lipid cell membrane and interact with membrane embedded electron transport proteins as well as menaquinone. *P. pasteri* KLE1280 is specific in its quinone requirement for growth requiring menaquinones but not ubiquinones. In hindsight, it is fortunate that a genetic screen was done before an attempt to identify the growth factor from *E. coli* by bioassay. The bioassay-driven purification proved highly challenging, as the spent supernatant of *E. coli* did not show consistent induction of *P. pasteri* KLE1280, even after concentration.

**Table 2.** Quinone specificity of *P. pasteri* KLE1280.

Compound	Growth	Source	Growth
Menaquinone 4	+	Ubiquinone 1	-
Menaquinone 5	-	Ubiquinone 2	-
Menaquinone 6	-	Ubiquinone 4	-
Menaquinone 7	-	Ubiquinone 8	-
Menaquinone 8	-	Ubiquinone 9	-
DHNA	+	Ubiquinone 10	-
Menadione	-	Media control	-

### *Genome analysis of* P. pasteri *KLE1280 for menaquinone synthesis pathway*

To get a better understanding of the general biology and specifically of the quinone biosynthesis of *P. pasteri* KLE1280, its genome was sequenced by The Genome Institute at Washington University. The annotated genome sequence is available from NCBI as JNOS01000000. The 16S rRNA sequence is available from NCBI as OP480832. Annotation was done using RAST [21] which involves two primary steps:

1. Identification of tRNA genes using tRNAscan-SE and rRNA genes using ‘search_for_rnas’ developed by Niels Larsen. Protein encoding genes are identified using GLIMMER3 to identify ORFs.
2. Functional assignment of ORFs using an in-house curated subsystem (collections of functionally related protein families) and a derived FIGfams (protein families) to assign protein functions.

**Figure 7.**
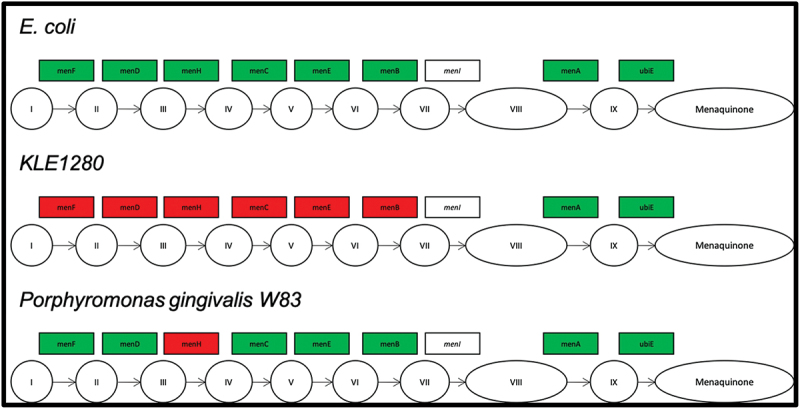
Genes involved in the menaquinone biosynthesis pathway identified in the genome sequence of *E. coli, P. pasteri* KLE1280, and *P. gingivalis* W83. Genes in filled green boxes have been identified in the genome sequence of the isolate, while red filled boxes have not been identified in the genome of the isolate.

The genome was searched for the presence of menaquinone pathway genes. ORFs for *menA* and *ubiE* were identified, while those for menF, menD, menC,*men H, menE*, and *menB* were not. The results of this analysis are shown in Figure 7. In contrast to *P. pasteri*, the genome of *P. gingivalis* W83 (AE015924.1) contains all of the recognized genes in the synthetic pathway except for gene *menH. MenH* was not identified in *P. gingivalis* by BLASTP using the gene from *E. coli*. It is likely present but not identifiable by BLAST.

These findings indicate that the genes in the *E. coli* menaquinone pathway that were essential to its ability to help the growth of *P. pasteri* were those leading to the synthesis of DHNA. *P. pasteri* possesses the two final genes in the menaquinone pathway that convert DHNA to menaquinone. Thus, the deletion analysis and quinone analyses are consistent with *E. coli* supporting growth of *P. pasteri* by secreting DHNA, which *P. pasteri* can convert to menaquinone.

### *Heme requirement for* P. pasteri *KLE1280*

**Figure 8.**
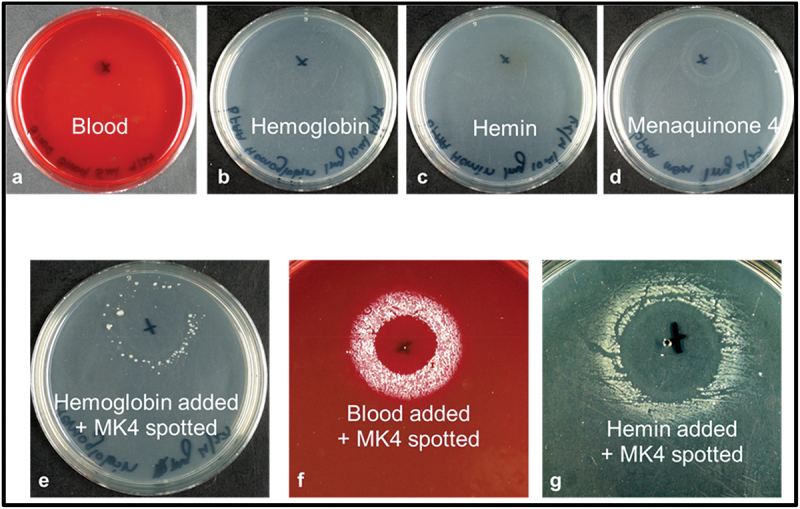
Heme requirement of *P. pasteri* KLE1280. The strain was spread evenly on FAA (without hemin) plates containing blood (**a**), hemoglobin (**b**), hemin (**c**), MK4 (**d**), hemoglobin and MK4 (**e**), blood and MK4 (**f**) and hemin and MK4 (**g**). *P. pasteri* KLE1280 grew only in presence of both MK4 and hemoglobin, blood or hemin (**e-g**).

Because members of the genus *Porphyromonas* require heme for growth [28], we examined the heme requirement for *P. pasteri* KLE1280. As shown in Figure 8, *P. pasteri* KLE1280 grew when a source of heme and MK4 (or DHNA) was supplied. Added hemin, blood, or hemoglobin fulfilled the heme requirement for growth. The heme requirement results from an incomplete heme biosynthetic pathway in *P. pasteri* and *P. gingivalis*.

### *Menaquinone pathway genome analysis of* Porphyromonas *and* Tannerella *oral taxa*

To determine if other strains of *P. pasteri*, or strains of other oral species in the genera *Porphyromonas* and *Tannerella*, had complete or incomplete menaquinone synthesis pathways, we examined 93 genomes available in the NCBI database, representing 11 oral taxa. The analysis found an operon, composed of *menBCDEF*, that was either present or absent in each genome. In the 5-gene operon, the gene order was always *menFDBCE*. The last two genes in the synthetic pathway, *menA* and *ubiE*, were present in all genomes but were scattered in the genomes as individual genes. The genome of *P. pasteri* type strain JCM 30531 and Forsyth strain F0450 lacked the operon for the early pathway as did strain *P. pasteri* KLE1280. The genomes of all strains of *Porphyromonas asaccharolytica, Porphyromonas uenonis* and *T. forsythia* contained the full pathway. *P. gingivalis* was unique in having some strains with and some strains without the complete pathway. Interestingly, among the 64 strains of *P. gingivalis*, 31% contained the full pathway while the others had incomplete pathways. *Porphyromonas catoniae, Porphyromonas endodontalis, Porphyromonas* sp. HMT-275, *Porphyromonas* sp. HMT-278, *Tannerella* sp. HMT-286 and *Tannerella* sp. HMT-808 had incomplete pathways. In *P. gingivalis* and *T. forsythia* genomes, each of the genes in the 5-gene operon shared 94–98% identity with those in genomes from other strains of these two species. However, the genes in the operon from *P. gingivalis* and *T. forsythia* are only distantly related, <73%, to homologous genes in the 5-gene operon from *P. asaccharolytica* and *P. uenonis*. It would therefore appear that this 5-gene operon is gained or lost through horizontal gene transfer rather than passed vertically. Results from this analysis are summarized in Table 3 and a full listing of the proteins identified for each of the 93 strains is available as supplementary Table S1.

**Table 3.** Menaquinone synthesis pathway gene summary.

Genus	Species	menF	menD	menC	menE	menB	menA	ubiE
*Porphyromonas*	*asaccharolytica*	100% (2/2)	100% (2/2)	100% (2/2)	100% (2/2)	100% (2/2)	100% (2/2)	100% (2/2)
*Porphyromonas*	*catoniae*	0% (0/2)	0% (0/2)	0% (0/2)	0% (0/2)	0% (0/2)	100% (2/2)	100% (2/2)
*Porphyromonas*	*endodontalis*	0% (0/1)	0% (0/1)	0% (0/1)	0% (0/1)	0% (0/1)	100% (1/1)	100% (1/1)
*Porphyromonas*	*gingivalis*	31% (20/64)	31% (20/64)	31% (20/64)	31% (20/64)	31% (20/64)	31% (20/64)	30% (19/64)
*Porphyromonas*	*pasteri*	0% (0/5)	0% (0/5)	0% (0/5)	0% (0/5)	0% (0/5)	100% (5/5)	100% (5/5)
*Porphyromonas*	*sp* . HMT-275	0% (0/1)	0% (0/1)	0% (0/1)	0% (0/1)	0% (0/1)	100% (1/1)	100% (1/1)
*Porphyromonas*	*sp* . HMT-278	0% (0/1)	0% (0/1)	0% (0/1)	0% (0/1)	0% (0/1)	100% (1/1)	100% (1/1)
*Porphyromonas*	*uenonis*	100% (2/2)	100% (2/2)	100% (2/2)	100% (2/2)	100% (2/2)	100% (2/2)	100% (2/2)
*Tannerella*	*forsythia*	100% (10/10)	100% (10/10)	100% (10/10)	100% (10/10)	100% (10/10)	100% (10/10)	100% (10/10)
*Tannerella*	*sp* . HMT-286	0% (0/2)	0% (0/2)	0% (0/2)	0% (0/2)	0% (0/2)	100% (2/2)	100% (2/2)
*Tannerella*	*sp* . HMT-808	0% (0/3)	0% (0/3)	0% (0/3)	0% (0/3)	0% (0/3)	67% (2/3)	100% (3/3)

Editor’s Note: Sp. Should not be in italics

### *Menaquinone growth requirements of* P. pasteri *and* P. gingivalis *strains*

As an experimental validation of the genomic analysis, we tested the ability of selected strains to grow with and without supplementation with menadione or DHNA. Four strains of *P. pasteri* and five strains of *P. gingivalis* were examined to determine how the identification of a complete menaquinone synthesis pathway within the genome correlated with the response to addition of menadione or DHNA to their growth media (Table 4).

**Table 4.** Menaquinone factor requirement for *P. pasteri* and *P. gingivalis* strains.

Species	Strain	Pathway	Ethanol^a^	Menadione^a^	DHNA^a^
*Porphyromonas pasteri*	KLE1280	Incomplete	-	+	+++
*Porphyromonas pasteri*	CCUG 66735 ^T^	Incomplete	-	+	+++
*Porphyromonas pasteri*	H37B19	Incomplete	-	-	+++
*Porphyromonas pasteri*	F0450	Incomplete	++	+++	+++
*Porphyromonas gingivalis*	ATCC 33277 ^T^	Incomplete	-/+	++	++
*Porphyromonas gingivalis*	F0568	Incomplete	-/+	-/+	+++
*Porphyromonas gingivalis*	F0570	Incomplete	-	++	+++
*Porphyromonas gingivalis*	W83	Complete	+++	+++	+++
*Porphyromonas gingivalis*	F0185	Complete	++	+++	+++

^a^Growth was categorized as: – no growth; + growth adjacent to spot; ++ growth within 2 cm of spot; and growth covering most of plate +++.

Among the seven strains tested that lacked a complete menaquinone pathway, all but one did not grow, or grew weakly, on media without added menadione or DHNA. Most of these strains were stimulated more by DHNA than menadione. The two *P. gingivalis* strains with the complete pathway grew strongly with or without addition of menadione or DHNA to their media.

## Discussion

It is advantageous for a growth factor-dependent bacterium to be near factor-producing neighbors as factor concentration decreases with diffusion distance. Our strategy of picking a small or slow growing colony satelliting a large colony is thus valuable for obtaining an auxotrophic bacterial isolate and its factor-producing helper bacterium. This strategy is consistent with the work of laboratories that used cross streaking methods to recover growth factor-dependent isolates [6–8,14,15,29,30]. Studies have shown that previously uncultured organisms will grow on nutrient medium only in the presence of other species from their environment [31,32]. While culture of growth factor-dependent bacteria in the presence of helper organisms is useful for some studies, many microbial experiments require the use of an organism grown axenically. Culturomics is a high throughput approach to getting such axenically growing bacteria, especially novel bacteria [33]. In the culturomics strategy, a large number of culture conditions are used, combined with matrix-assisted laser desorption/ionization-time-of-flight (MALDI-TOF) mass spectrometry to identify novel previously uncultivated bacteria. Culturomics is certainly a promising approach to identifying members of the human microbiome in different body sites for investigators with access to a MALDI-TOF instrument and who have built up a large reference library of dual microbial mass spectrum fingerprints and 16S rRNA identifications. However, in the culturomics approach, colonies identified by MALDI-TOF must grow axenically in subculture on the medium from which they were cultured and so this approach would lose the dependent bacteria captured in our approach.

Growth factor identification is greatly facilitated if a deletion mutant library is available or can be produced for the helper bacteria as this allows for a genetic screen of pathways or genes involved in growth factor production. Once pathways or genes are identified, metabolites can be screened for stimulation of the auxotrophic bacteria to identify the growth factor. This approach allowed the identification of DHNA as a growth factor for *P. pasteri* KLE1280 and additional *Porphyromonas* and *Tannerella* species. DHNA is a valuable growth factor that can be added to media to facilitate recovery of fastidious bacteria not recovered on conventional media lacking DHNA.

The correlation between lack of a complete menaquinone synthesis pathway in the genome sequence and a growth requirement *in vitro* for menadione or DHNA was found for most of the *P. pasteri* and *P. gingivalis* strains examined (Table 4). The exception was *P. pasteri* strain F0450, which has been passaged since 2013 and appears to have adapted to laboratory culture, as it no longer showed the dependent phenotype. In efforts to identify growth factors required by dependent isolates, a recurring problem is that upon repeated passage in the laboratory, the dependent isolate loses its dependent phenotype. Preservation of low passage stocks is important for maintaining a strain with an important phenotype.

The role of quinones in an electron transport chain is most well known in aerobic organisms where electrons and hydrogen are transferred to O_2_, producing H_2_O. The role of menaquinones in anaerobic bacteria is also important so that they can respire using other electron acceptors such as nitrate, sulfate Fe^III^, or fumarate. A key role of menaquinone in *Porphyromonas* is the reduction of fumarate to succinate using the enzyme fumarate reductase (quinol) flavoprotein [34]. *P. pasteri* strains KLE1280, F0450 and JCM 30531 all possess fumarate reductase (quinol) flavoprotein (*frdA)* proteins KDU78448, EJU17319, and GGM57237, respectively. The gene appears to be present in all *P. gingivalis* strains and *Porphyromonas* species examined. The pool of fumarate available for reduction likely comes from the action of aspartate ammonia-lyase on the intracellular pool of aspartate as the *Porphyromonas* species are proteolytic rather than saccharolytic.

While all *Porphyromonas* species appear to need some form of exogenous heme for growth, we validated the heme requirement for *P. pasteri* KLE1280 (Figure 8). This strain requires hemin, hemoglobin or blood for growth. In the well-studied *P. gingivalis*, the heme biosynthesis pathway is incomplete [35]. *Porphyromonas* species have several mechanisms for acquiring heme and iron from their host [28,35]. The growth factors required for *P. pasteri* KLE1280 to grow on a general commercial medium are exogenous heme and DHNA.

It was a surprise to find an apparently essential component of the electron transport chain in the growth of a bacterium missing from the genome. The above results suggest that *P. pasteri* KLE1280 lacks the ability to make its own menaquinones, yet apparently a functioning electron transport chain is essential for this organism. Given that there are species amongst the *Porphyromonas* and *Tannerella* that are missing these essential genes, the hypothesis is that these organisms occupy niches close to other bacteria in the oral cavity. This would explain the presence of *Porphyromonas* in a corncob structure with other bacteria within the dental plaque matrix, presumably to obtain these growth factors [36]. Thus, the presence of such structures in the dental plaque could be a necessity for such organisms that have lost the ability to make a growth factor to be close to organisms producing these growth factors.

*P. pasteri* is one of the 12 most abundant bacteria in the oral cavity and the second most abundant in saliva [13]. In several studies examining the salivary microbiome or oral rinses, the relative abundance of *P. pasteri* was positively associated with oral health while lower relative abundance was associated with periodontal disease [37–39], Sjogren’s syndrome [40], and oral squamous cell carcinoma [41]. In contrast to its role in oral health, in cystic fibrosis, it is associated with a decline in lung function [42]. *P. pasteri* is not only abundant in all nine of the oral sites studied in human microbiome [13] but it is also present in the vaginal microbiome where it has been isolated and a genome sequence produced (*P. pasteri* strain KA00683 Table S1) [43]. Considering its oral abundance, oral health association, and possible association with disease in other body sites, *P. pasteri* appears to be an important bacterial species that has been remarkably understudied. Future studies of this species should be greatly facilitated by knowing that its isolation and axenic culture depend on, or are enhanced by, culture in media containing DHNA.

### Conclusions

Picking a small or slow growing colony satelliting a large colony is a valuable strategy for obtaining an auxotrophic bacterial isolate and its factor producing helper bacterium. Growth factor identification is greatly facilitated if a deletion mutant library is available or can be produced for the helper bacteria as this allows for a genetic screen of pathways or genes involved in growth factor production. Once pathways or genes are identified, metabolites can be screened for stimulation of the auxotrophic bacteria to identify the growth factor. This approach allowed the identification of DHNA as a growth factor for *P. pasteri* KLE1280 and additional *Porphyromonas* and *Tannerella* species. DHNA is a valuable growth factor that can be added to media to facilitate recovery of fastidious bacteria not recovered on conventional media lacking DHNA.
